# Putting Plasticity in Its Place

**DOI:** 10.3389/fnins.2012.00110

**Published:** 2012-07-13

**Authors:** Josef H. L. P. Sadowski, Jack R. Mellor

**Affiliations:** ^1^MRC Centre for Synaptic Plasticity, School of Physiological and Pharmacology, University of BristolBristol, UK

The dynamics of synaptic plasticity and the encoding of spatial information have been extensively studied in the hippocampus. Yet few attempts have been made to investigate how the two combine to produce the most striking emergent function of this system, the cognitive map (Toleman, 1948).

Here, Bush et al. present a neural network model describing how a cognitive map based on empirical findings from hippocampal electrophysiology could function in order to encode space and enable sequence learning. The model demonstrates how both functions could be accommodated within the same network architecture and using the same learning rule (Bush et al., 2010).

The network employed by the authors is based on the recurrently connected CA3 region of the hippocampus, where individual “place cells” fire maximally at specific locations within one, two, and three-dimensional space (O'Keefe and Dostrovsky, 1971; O'Keefe, 1976; Hayman et al., 2011). Connection strengths are fully modifiable and are governed by spike-timing dependent plasticity (STDP) rules based on empirical findings from hippocampal cultures (Bi and Poo, 1998; Debanne et al., 1998; Wang et al., 2005). Here, the timing of pre- and post-synaptic activity is crucial in determining the magnitude and direction of synaptic plasticity, in contrast to purely rate-coded synaptic dynamics. In addition, changes in synaptic weights are dynamically modulated by ongoing theta-coded activity dictated by a phenomenological phase precession model (O'Keefe and Recce, 1993; Huxter et al., 2008).

When the network experiences neural activity corresponding to a series of shuttle runs across a square arena, heteroassociative connections between activated place cells become potentiated, cells with overlapping place fields develop strong bidirectional connections and asymmetric connections between cells against the direction of motion become depressed. The elevated connectivity of the activated ensemble resembles the broad development of a “cognitive map” with bidirectional connections providing detailed information about the relative proximity of individual place fields. The firing patterns of cells with overlapping place fields satisfy the STDP rule and enable connections between such cells to become potentiated, as demonstrated in slices (Isaac et al., 2009). The biasing of asymmetric connection weights toward the last direction of motion enables the encoding of recent trajectories or episodes. Thus the requirements for spatial encoding and sequence learning can be fulfilled simultaneously. The authors note that if there was only a single direction of motion, the connections between the place cells would become purely asymmetric and the cognitive map would thus remain undeveloped.

During simulated random exploration of an arena, a similar pattern of bidirectional connectivity develops between place cells encoding proximal place fields, with local asymmetric connections reflecting fragments of recent trajectories. Repeated traversals of a one dimensional route in a single direction through such a learned environment does not alter the gross structure of the map, but profoundly alters the connections of place cells that lie on the route. As before, connections following the direction of travel are significantly potentiated but those against it are depressed, suggesting information concerning recent trajectories can be embedded in the cognitive map.

The model goes some way to supporting *in vivo* and *in vitro* data demonstrating the role of synaptic plasticity within the of development of the cognitive map and the encoding of episodic memory; it also prompts a number of predictions as to when and where plasticity may be induced during these processes (Sadowski et al., 2011). However, these predictions are perhaps limited to the experimental context within which their STDP rule is rooted – that being data gleaned primarily from hippocampal cultures rather than acute brain slices (Buchanan and Mellor, 2010). The model is used to explore the consequences of dynamic plasticity modulation by theta-coded activity, yet other dynamic factors, most notably cholinergic tone, are also known to be powerful modulators of synaptic plasticity and may have a crucial role in tuning the development of the cognitive map.

One unexplored aspect of the paper relates to the “offline” dynamics of a network when such patterns of connectivity have been established. The phenomenon of place cell replay has been observed during *in vivo* hippocampal recordings, where sequences of place cell activity are recapitulated during sleep or rest following behavior (Skaggs and McNaughton, 1996). It is postulated that replay may facilitate the long term consolidation of episodic memory by driving correlated activity in other brain regions such the neocortex (Walker and Stickgold, 2010). In theory the potentiated asymmetric connections between place cells involved in recent route traversals would make it more likely for them to be reactivated in the same sequence during the post-encoding phase. Forward and reverse replay of place cell firing patterns have been observed in behaving rats (Foster and Wilson, 2006; Diba and Buzsaki, 2007), yet the model by Bush et al. does not provide as obvious an explanation for reverse replay than forward replay. The involvement of replay would allow learned sequences to become consolidated in the cortex, but it suggests that the consolidation of the cognitive map may be more piecemeal, if indeed it occurs at all.

Overall, the paper demonstrates how computational modeling can elegantly combine parallel strands of hippocampal electrophysiology and make defined predictions about how behavior might shape connectivity and therefore the function of neural networks. An integrated *in vivo* and *in vitro* approach is necessary to test such predictions; recording from place cells during behavior and investigating the consequences of such activity on connectivity. In addition, robust empirical data regarding the influence of neuromodulators on STDP must be acquired in order to improve the physiological relevance of this promising model of plasticity and the cognitive map.
